# Pathway for Biodegrading Microcystin-YR by *Sphingopyxis* sp. USTB-05

**DOI:** 10.1371/journal.pone.0124425

**Published:** 2015-04-28

**Authors:** Huimin Xu, Huasheng Wang, Qianqian Xu, Le Lv, Chunhua Yin, Xiaolu Liu, Hongwu Du, Hai Yan

**Affiliations:** School of Chemistry and Biological Engineering, University of Science and Technology Beijing, Beijing, 100083, PR China; Mount Allison University, CANADA

## Abstract

Harmful cyanobacterial blooms in waters have become a global environmental problem, this mainly due to the production and release of various microalgal toxins, in which microcystins (MCs) are distributed widely. Here, we focused on the study of a typical form of microcystins called microcystin-YR (MC-YR). It was found that initial 14.8 mg/L of MC-YR could be completely eliminated within 10 hr by the crude enzymes (CEs) of *Sphingopyxis* sp. USTB-05, a promising bacterial strain we isolated and identified in our previous study. During the enzymatic biodegradation of MC-YR with time course, the peaks of two intermediate and two final products were observed on the profiles of HPLC at the wavelengths of 238 nm and 230 nm, respectively. Based on the analysis of *m/z* ratios of MC-YR and its four products by LC-MS/MS, we suggested that at least four enzymes were involved in the biodegradation of MC-YR by *Sphingopyxis* sp. USTB-05. The first enzyme microcystinase converted cyclic MC-YR to linear MC-YR as the first product. Then the second enzyme serine protease was found to cleave the target peptide bond between alanine (Ala) and tyrosine (Tyr) of linearized MC-YR, producing a tetrapeptide and a tripeptide as second products, which were Adda-Glu-Mdha-Ala and Tyr-Masp-Arg, respectively. Next, the third enzyme peptidase converted the tetrapeptide of Adda-Glu-Mdha-Ala to Adda. And the fourth enzyme cleaved the tripeptide of Tyr-Masp-Arg to produce Tyr and dipeptide (Masp-Arg), which has never been reported. These findings will help us better understand the biodegradation pathway of MC-YR by *Sphingopyxis* sp. USTB-05.

## Introduction

With the development of industry and agriculture, a great amount of wastewater containing nitrogen and phosphorus has been discharged into rivers and lakes. Driven by the global warming, harmful cyanobacterial blooms have become more ubiquitous. About 70% of cyanobacterial blooms produce toxins, in which microcystins (MCs) are the most commonly detected and widely distributed around the world [1]. MCs have been studied extensively, not only for their ability to cause acute poisonings [2,3], but also for their existence in drinking water which can strongly induce liver tumors despite a low concentration [4]. MCs are so poisonous [5] that they have severely threaten the security of water resources and the stability of aquatic ecosystems.

MCs are compounds of monocyclic heptapeptide containing amino-9-methoxy-2,6,8-trimethyl-10-phenyldeca-4(E),6(E)-dienoic acid (Adda) that is responsible for the hepatotoxicity of molecules. More than 70 variants of MCs have been isolated and identified in which microcystin-RR (MC-RR), microcystin-LR (MC-LR) and microcystin-YR (MC-YR) are considered to be the most common and dangerous variants [6], [7,8]. However, they can be readily biodegraded by a range of aquatic bacteria [9–15], such as *Pseudomonas aeruginosa* [16], *Burkholderia* sp. [17]. In 2010, we isolated and identified a promising bacterial strain of *Sphingopyxis* sp. USTB-05 by the analysis of 16S rDNA (GenBank database under accession number: EF607053). Initial MC-RR of 42.3 mg/L could be completely biodegraded by *Sphingopyxis* sp. USTB-05 within 36 hr, and by its crude enzymes (CEs) within 10 hr, respectively [18]. Furthermore, initial MC-LR of 21 mg/L could also be totally removed within 20 hr and 10 hr by USTB-05 and its CEs, respectively [19], indicating that *Sphingopyxis* sp. USTB-05 indeed has a strong ability in the biodegradation of MCs.

Enzymatic pathway for biodegrading MC-LR and MC-RR by *Sphingomonas* sp. have been widely studied and at least three enzymes, including microcystinase, serine protease and peptidase, were found to be involved in the sequential biodegration of MC-LR and MC-RR [10], [18,19]. The first enzyme, microcystinase, is considered to be the most important one, as it can turn the highly stable cyclic MC-LR or MC-RR into linear structure by the hydrolytic cleavage between Adda and Arg, leading to a 160-fold reduction in toxic activity of the parent MC-LR or MC-RR. The second enzyme, serine protease, is responsible for converting linearized MC-LR or MC-RR into the tetrapeptide of NH_2_-Adda-Glu (iso)-Mdha-Ala-OH. The third enzyme, peptidase, plays its part by cleaving the tetrapeptide to produce Adda [9], [11,12], [18,19]. Although the biodegradation is found to be the most efficient method for removing MCs, and the enzymatic pathways of MC-LR and MC-RR have been widely studied, little information is provided on the biodegradation of MC-YR which contains a more complex Tyr in the ring structure of heptapeptide, and is also a typical type of MCs produced by cyanobacteria is potent hepatotoxin and tumor promoter. Not only destroy the balance of aquatic ecosystem, but pose a threaten to the survival of humans and animals. Because MC-YR is chemically stable compounds, conventional drinking water treatments have limited efficacy in removing MC-YR.

Here the enzymatic biodegradation products of MC-YR by CEs of *Sphingopyxis* sp. USTB-05 were studied. Two intermediate and two final products were observed on the profiles of HPLC at the wavelengths of 238 nm and 230 nm, respectively. It was indicated that at least four enzymes were involved in the biodegradation of MC-YR by *Sphingopyxis* sp. USTB-05. The first enzyme microcystinase converted cyclic MC-YR to linear MC-YR as the first product. Then the second enzyme serine protease was found to cleave the target peptide bond between Ala and Tyr of linearized MC-YR, producing a tetrapeptide and a tripeptide as the second products, which were Adda-Glu-Mdha-Ala and Tyr-Masp-Arg, respectively. Next, the third enzyme peptidase converted the tetrapeptide of Adda-Glu-Mdha-Ala to Adda. And the fourth enzyme cleaved the tripeptide of Tyr-Masp-Arg to produce Tyr and dipeptide (Masp-Arg), which has not been previously found. These findings are very important in both the basic research and the removal of MC-YR from natural water source.

## Materials and Methods

### Chemicals

Standard MC-YR (≥95% purity, Molecular formula: C_52_H_72_N_10_O_13_, MW: 1045.5, Enzo Science Inc. USA.) and Tyr (≥95% purity, Molecular formula: C_9_H_11_NO_3_, MW: 181.2, Sigma Chemical Co., Ltd., USA) were purchased and stored at -20°C until use. Chromatographic grade methanol and acetonitrile were used to prepare the mobile phase in HPLC and LC-MS/MS analyses. All other chemicals used in this study were analytical grade except those specified by the kits.

### Bacterial strain and cultural conditions

A bacterial strain of *Sphingopyxis* sp. USTB-05 we previously isolated and identified was used to biodegrade MC-YR [18]. The bacterial strain was cultured at 30°C with the shake rate of 200 r/min. The culture medium contained MgSO_4_·7H_2_O (1.0 g), KH_2_PO_4_ (0.5 g), K_2_HPO_4_ (4.0 g), NaCl (1.0 g), CaCl_2_ (20.0 mg), FeSO_4_ (5.0 mg), ZnCl_2_ (5.0 mg), MnCl_2_·4H_2_O (5.0 mg), CuCl_2_ (0.5 mg), glucose (15.0 g), and yeast (1.5 g) per 1000 mL at initial pH 7.2. The prepared medium was sterilized at 121°C for 20 min and then *Sphingopyxis* sp. USTB-05 was inoculated into the medium for culture.

### Preparation of the crude enzymes (CEs)

At logarithmic growth phase of the third day, the culture solution of *Sphingopyxis* sp. USTB-05 was centrifuged at 8,000 r/min for 20 min, and then the supernatant was decanted. The pelleted cells were washed three times with 50 mmol/L phosphate buffer solution (PBS, pH 7.0). The harvested cells were resuspended in 20 mL PBS and disrupted using an ultrasonic disruptor with an output power of 600 W for 15 min at 4°C. The cell debris was removed by centrifugation at 12,000 r/min for 30 min. The supernatant was collected, and after handling by 3500 MWCO dialysis bag small molecules such as Tyr was removed, it was used as CEs in the enzymatic biodegradation of MC-YR and its protein profile was determined using sodium dodecyl sulfate-polyacrylamide gel electrophoresis (SDS-PAGE) on 12% polyacrylamide gel. The concentration of protein in CEs was measured as 350 mg/L by the method of Bradford (1976) using bovine serum albumin as the standard.

### Enzymatic biodegradation of MC-YR

The total biodegradation reaction volume was 5 mL containing 14.8 mg/L MC-YR and 103.6 mg/L protein in PBS in a 10 mL centrifuge tube. The reaction was at 30°C with the shake rate of 200 r/min. The samples of 0.5 mL for each were taken at 0, 1.5, 3, 5, 10 and 24 hr, respectively, and then 0.05 mL of concentrated hydrochloric acid (36%, *v/v*) was added to each sample to stop the reaction. All samples were centrifuged at 12,000 r/min for 10 min, and then the supernatant was used to measure MC-YR and its products with HPLC.

In order to identify the products of MC-YR, the samples of 0.5 mL were passed through a C_18_ solid-phase extraction cartridge (Waters, OASISTMHLB, USA, 30 mg/mL). Methanol of 0.5 mL was used to elute the products of MC-YR with the rate of 1 mL/min. The elution was used to measure the mass/charge (*m/z*) of products with LC-MS/MS (API-3000, Applied Biosystems, USA).

### Analysis of MC-YR and its products

MC-YR and its enzymatic biodegradation products were firstly measured using a HPLC system (Shimadzu LC-10ATVP, Shimadzu Co., Ltd., Japan) with a ultraviolet (UV) Diode Array Detector at 238 nm and 230 nm using a Agilent TC-C_18_ column (4.6 mm×250 mm) (Agilent, 1200 series, USA). The mobile phase were 35% (*v/v*) acetonitrile water solution containing 0.05% (*v/v*) of trifluoroacetic acid at 238 nm that is the maximum absorption peak for Adda, and 1% (*v/v*) methanol water solution containing 8.5 mmol/L sodium acetate trihydrous at 230 nm that is the maximum absorption peak for Tyr, respectively. The flow rate was 1 mL/min and the injection amount was 20 μL.

Mass spectral (MS) analysis were performed in positive and negative ion electrospray mode on the liquid chromatogram-mass spectrum (LC-MS) (3200 Q TRAP, Applied Biosystems, USA). An Agilent TC-C_18_ analytica column from Waters Corp. (USA) was used in the chromatographic measurement in LC-MS analysis. For MS detection, precursor ions for samples and internal standards were determined from MS obtained during infusion into the mass spectrometer (3200 Q TRAP, Applied Biosystems, USA). Using an electrospray ionization (ESI) source, the mass spectrometer was operated in the positive and negative ionization mode with the collision gas off. ESI conditions were as follows: curtain gas = 15, ionspray voltage = 5000, temperature = 450, ion source gas 1 (N_2_) = 70, ion source gas 2 (N_2_) = 50, declustering potential = 100, entrance potential = 10.

## Results

### Enzymatic kinetics of MC-YR biodegradation by CEs of *Sphingopyxis* sp. USTB-05

**Fig 1:**
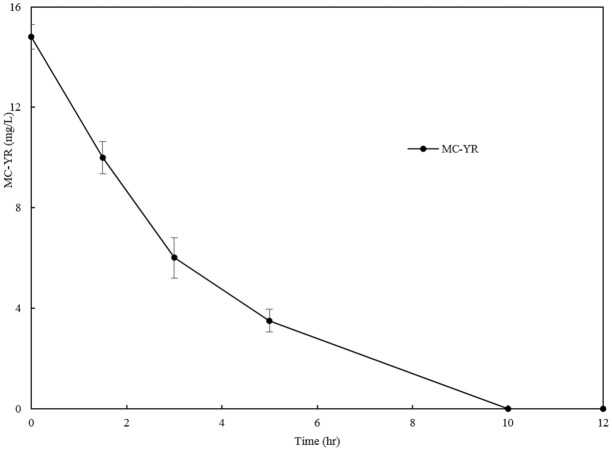
Biodegradation kinetics of MC-YR catalyzed by CEs of USTB-05.

Initial MC-YR of 14.8 mg/L rapidly decreased and was completely removed within 10 hr by CEs of *Sphingopyxis* sp. USTB-05 containing protein of 103.6 mg/L (Fig 1), and the average biodegradation rate of MC-YR was 1.5 mg/L per hour, demonstrating that CEs of *Sphingopyxis* sp. USTB-05 indeed has a strong ability in the biodegradation of MC-YR.

### Products of MC-YR measured at the wavelength of 238 nm on HPLC

**Fig 2:**
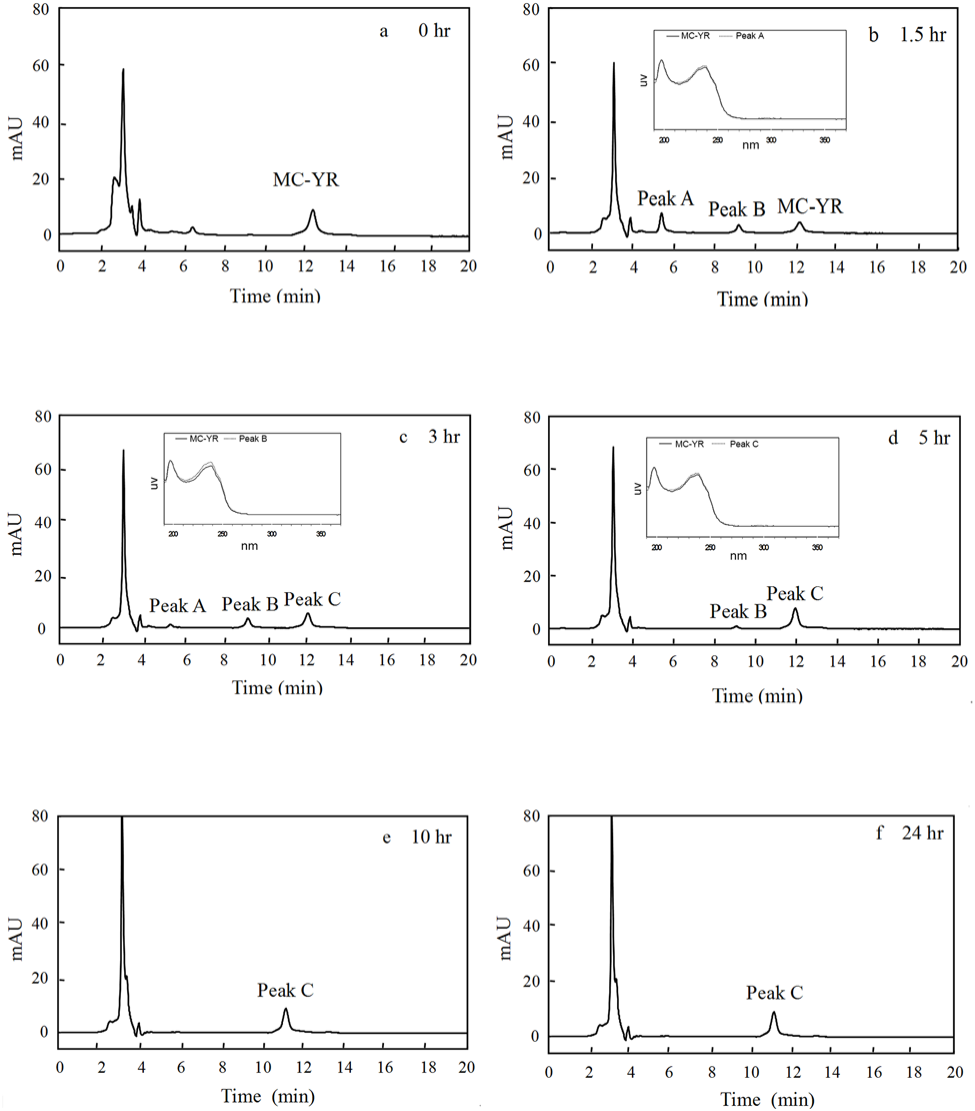
Enzymatic biodegradation of MC-YR by CEs of USTB-05 with time course and the scanning profiles of MC-YR and its products at the ultraviolet wavelength from 200 nm to 370 nm.

As shown in Fig 2a, the peak of MC-YR at the retention time of 12.1 min decreased over time. However, two new peaks of A and B at the retention time of 5.5 and 9.2 min appeared at 1.5 hr (Fig 2b). At 3 hr, peak A decreased, while peak B increased, and a new peak C appeared at the retention time of 12.1 min (Fig 2c). At 5 hr, peak A disappeared and peak B declined, but peak C increased (Fig 2d). After 5 hr, only peak C maintained and kept constant until at 24 hr (Fig 2e and 2f). The scanning absorbance profile of peak A, B or C were very similar to that of MC-YR in the wavelength from 200 nm to 370 nm (Fig 2b and 2d), and the maximum absorbance of MC-YR and its three products of peak A, B and C were all at 238 nm or so. Fig 2 indicated that CEs of USTB-05 has the enzymatic activity to catalyze MC-YR, and two intermediate and a final products were clearly observed over time on the profile of HPLC.

### Products of MC-YR measured at the wavelength of 230 nm on HPLC

**Fig 3:**
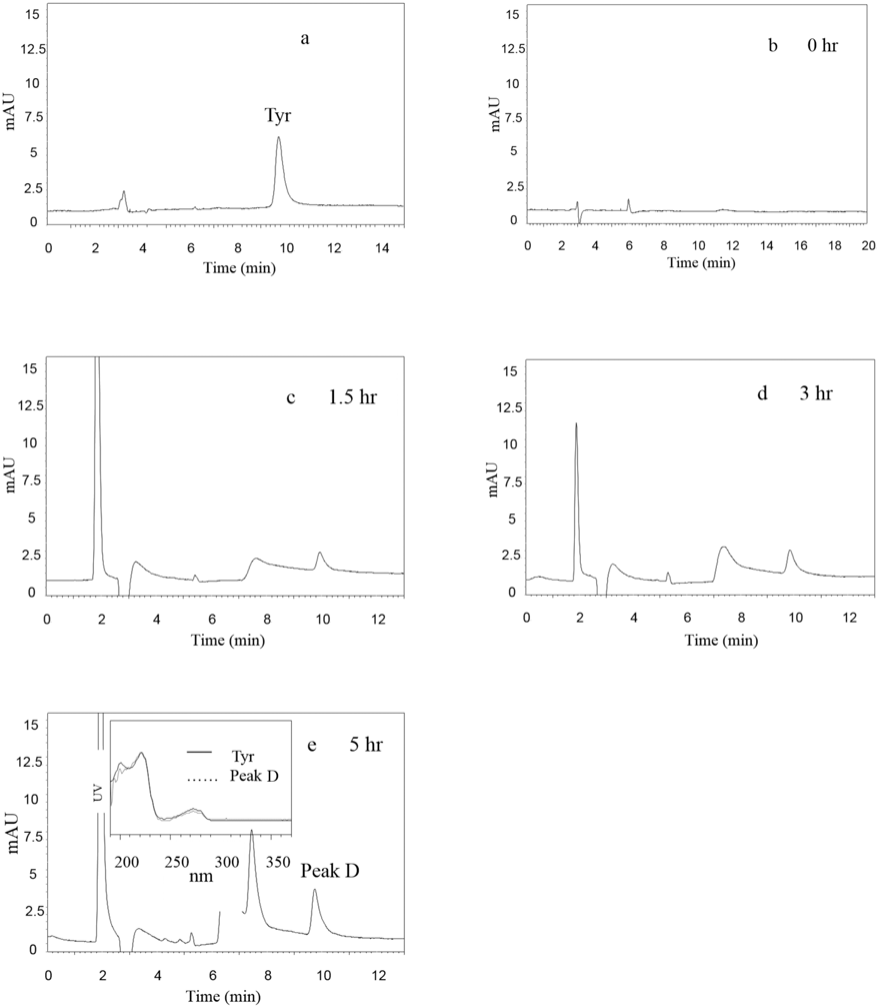
Enzymatic biodegradation of MC-YR by CEs of USTB-05 with time course and the scanning profiles of Tyr and its product at the ultraviolet wavelength from 200 nm to 370 nm.

As shown in Fig 3a, the standard Tyr peak was at the retention time of 9.7 min. At 0 hr, no peak was found at the retention time of 9.7 min, indicating that no Tyr was found in the initial reaction solution with CEs and MC-YR in PBS (Fig 3b). However, a new peak D at the same retention time of Tyr appeared and gradually increased from 1.5 to 5 hr (Fig 3c and 3e). Furthermore, the scanning absorbance profile of peak D was the same as that of Tyr, and both of their maximum absorbance was at 230 nm (Fig 3e). Fig 3 showed that a product of MC-YR catalyzed by CEs of USTB-05 may be Tyr.

### Mass/charge (*m/z*) ratios of MC-YR and its products

Mass/charge (*m/z*) ratios of MC-YR and its four products were shown in following. Based on the retention time and UV chromatogram in the HPLC profiles, peaks with retention times of 12.1, 5.5, 9.2 and 12.1 min at 238 nm were indicated as MC-YR, product A, product B and product C, respectively, and the peak with a retention time of 9.7 min at 230 nm was indicated as product D.

**Fig 4:**
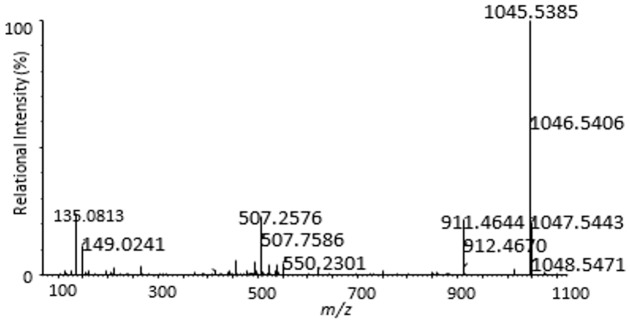
Liquid chromatogram-mass spectrum (LC-MS) profile of MC-YR.

The mass spectral analysis of MC-YR revealed a major ion at *m/z* 1045.5, corresponding to the [M+H]^+^ protonated molecular ion (Fig 4 and Table 1).

**Table 1:** Liquid chromatogram-mass spectrum (LC-MS) protonated molecular ion for MC-YR.

*m/z*	Identity
1046.5	M+H

**Fig 5:**
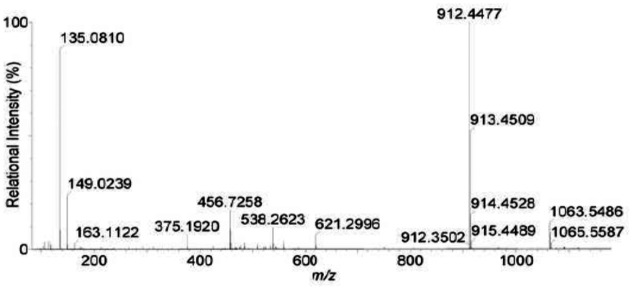
Liquid chromatogram-mass spectrum (LC-MS) profile of product A.

As shown in Fig 5 and Table 2, product A showed a protonated molecular ion at *m/z* 1063 and a main peak at *m/z* 862.4. The ion at *m/z* 1063 indicated that product A was linearized MC-YR ([M**+**H_2_O**+**H]^**+**^, M**+**18). The peak at *m/z* 912.4 (M+18–151) corresponded to the loss of the terminal phenylethymethoxy group (MW: 135) and the amino NH_2_ group (MW: 16) from Adda. The presence of this peak evidenced that the linearized MC-YR product contained N-terminal Adda. Besides, the presence of carboxy-terminal arginine (Arg) was demonstrated by protonated ion *m/z* 621.3 [Mdha-Ala-Tyr-Mesp-Arg-OH+H]^**+**^ and *m/z* 538.3 [Ala-Tyr-MeAsp-Arg-OH+H]^**+**^ (Fig 5 and Table 2).

**Table 2:** Liquid chromatogram-mass spectrum (LC-MS) protonated molecular ion for product A.

*m/z*	Identity
1063.5	Mass(M)+H_2_O+H
912.4	M+H_2_O+H-PhCH_2_CHOCH_3_-NH_2_
621.3	Mdha-Ala-Tyr-Masp-Arg-OH+H
538.3	Ala-Tyr-Masp-Arg-OH+H
375.2	Adda (-PhCH_2_CHOCH_3_)-Glu-Mdha+OH
135.0	PhCH_2_CHOCH_3_+H

**Fig 6:**
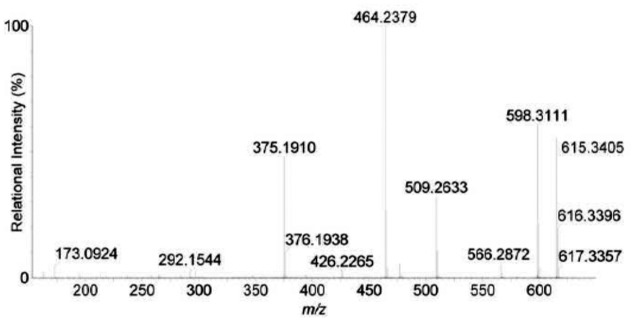
Liquid chromatogram-mass spectrum (LC-MS) profile of product B.

As shown in Fig 6 and Table 3, the protonated molecular ion of product B was detected at *m/z* 615.3 [M+H]^**+**^ resulted from the loss of tripeptide Tyr-MeAsp-Arg from linear MC-YR. The peak at *m/z* 464.2 [M+H-151]^**+**^ corresponded to the loss of the terminal phenylethylmethoxy group (MW: 135) and the amino NH2 group (MW: 16) from Adda, and the ions at *m/z* 509 was caused by the loss of the Adda amino group from the proposed parent compound. So, it was confirmed that product B was a tetrapeptide of Adda-Glu-Mdha-Ala (*m/z* 614) and another product was a tripeptide of Tyr-MeAsp-Arg (*m/z* 466).

**Table 3:** Liquid chromatogram-mass spectrum (LC-MS) protonated molecular ion for product B.

*m/z*	Identity
615.3	M+H (Adda-Glu-Mdha-Ala-OH+H)
598.3	M (–NH_3_)+H
566.3	Adda (–NH_2_–MeOH)-Glu-Mdha-Ala+H
509.3	Adda (–NH_2_)-Glu-Mdha+H
464.2	M (–PhCH_2_CHOMe–NH_2_) + H
426.2	Adda (–NH_2_)–Glu+H

**Fig 7:**
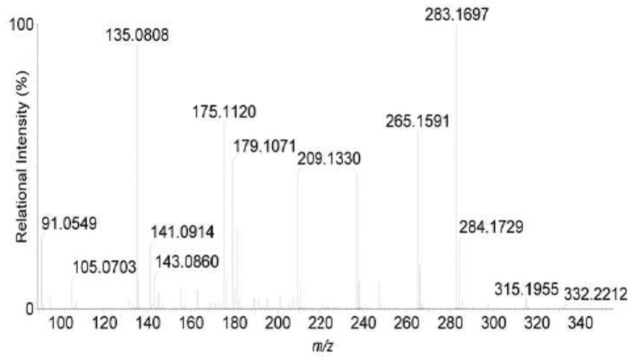
Liquid chromatogram-mass spectrum (LC-MS) profile of product C.

Fig 7 and Table 4 showed that the protonated molecular ion of product C was detected at *m/z* 332.2, *m/z* 315.2 and *m/z* 135.1, which was related to M+H, the loss of the amino NH2 group (MW: 16) from Adda and the PhCH2CHOMe part of Adda, respectively. Therefore, it was inferred that product C was Adda (*m/z* 331).

**Table 4:** Liquid chromatogram-mass spectrum (LC-MS) protonated molecular ion for product C.

*m/z*	Identity
332.2	M+H
315.2	M+H–CH_3_
179.1	M (–PhCH_2_CHOMe–NH_3_) + H
135.1	PhCH_2_CHOMe

**Fig 8:**
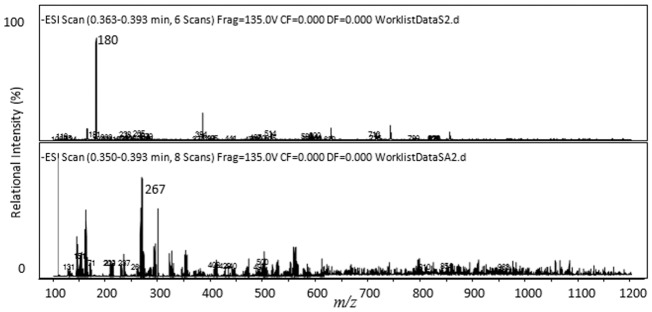
Liquid chromatogram-mass spectrum (LC-MS) profile of product D.

The MS spectrum for product D was shown in Fig 8 and Table 5, the protonated molecular ion of product D was detected at *m/z* 180 [M-H]^**-**^ resulted from the loss of tripeptide MeAsp-Arg and the the amino NH_2_ group (MW: 16) from tripeptide Tyr-MeAsp-Arg, and the ions at *m/z* 267 was caused by the loss of the Tyr from the proposed parent compound. Therefore, product D was proved to be Tyr (*m/z* 181).

**Table 5:** Liquid chromatogram-mass spectrum (LC-MS) protonated molecular ion for product D.

*m/z*	Identity
180	M–H
267	M–H

## Discussion

Harmful cyanobacterial blooms have become a growing environmental problem worldwide in natural waters. MCs are chemically stable compounds and conventional drinking water treatments have limited efficacy in removing MCs produced by harmful cyanobacteria. In contrast, biodegradation is found to be the most efficient methods to remove MCs with low cost, high safety, and is also conductive to ecological restoration. According to the previous reports, both microbial population [9], [20–23] and a single bacterial strain [9], [10], [13], [15–17], [24] were all testified to be capable of biodegrading MCs. *Sphingopyxis* sp.USTB-05 previously isolated from sediment of Dianchi Lake in China was a promising bacterial strain for the efficient biodegradation of MC-LR and MC-RR, and the biodegradation genes were also successfully cloned and expressed [18,19]. Although the biodegradation pathway and molecular mechanism of MCs have been widely studied, researches were mostly focused on MC-LR and MC-RR [10], [15–17], while less information was available on MC-YR that is also a dominant type of MCs in natural water body [25–27].

As for the biodegradation pathway and molecular mechanism of MC-LR and MC-RR, Shimizu et al. (2012) have reported at least three enzymes are involved in MC-LR biodegradation by *Sphingomonas* sp. strain. The first enzyme, can cleave the Adda-Arg peptide bond in MC-LR to produce the first product. After the cyclic structure is opened, the second enzyme cleave the Ala-Leu peptide bond in linear MC-LR to produce a tetrapeptide as the second product. And then, the third enzyme divide tetrapeptide into Adda as the third product. Based on the successful isolation and identification of *Sphingopyxis* sp. USTB-05 for the biodegradation of MC-RR [28], we have continuously investigated the genes and enzymes of *Sphingopyxis* sp. USTB-05. It was found that *Sphingopyxis* sp. USTB-05 possessed at least three enzymes involved in biodegradation of MC-LR and MC-RR, and three biodegradation genes have been firstly cloned and expressed [18,19], [28,29]. Compared with MC-RR and MC-LR, MC-YR is more complicated in structure containing a benzene ring of Tyr, which could also be rapidly biodegraded by CEs of *Sphingopyxis* sp. USTB-05. According to our study, initial 14.8 mg/L of MC-YR could be completely eliminated within 10 hr (Fig 1). In order to investigate the biodegradation pathway of MC-YR, the ring structures of both Adda and Tyr in MC-YR were traced, and four biodegradation products of MC-YR were successfully found over time at the wavelength of 238 nm and 230 nm on the HPLC profiles (Fig 2 and 3), respectively.

**Fig 9:**
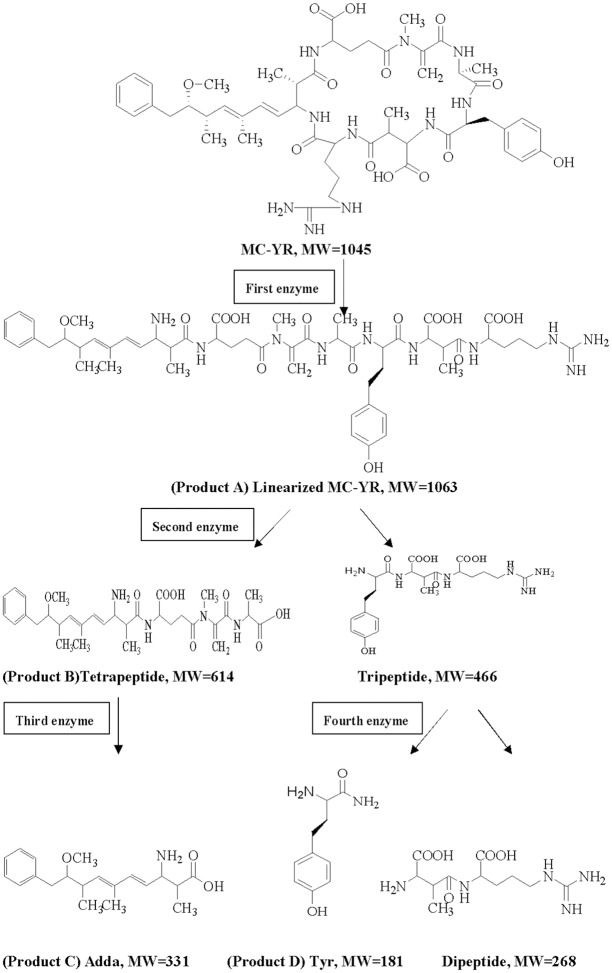
Proposed biodegradation pathway of MC-YR by Sphingopyxis sp. USTB-05.

Successful analysis of mass/charge (*m/z*) ratios of MC-YR and its four products (Figs 4–8) provided evidences for the biodegradation pathway of MC-YR by *Sphingopyxis* sp. USTB-05 (Fig 9), which was very similar to that of MC-RR and MC-LR. The first enzyme microcystinase was active in cleaving the target peptide bond between Adda and Arg of MC-YR (*m/z* 1045) (Fig 4) and converting cyclic MC-YR to linear MC-YR (*m/z* 1063) (Fig 5) as the first product (product A). Then the second enzyme serine protease was found to cleave the target peptide bond between Ala and Tyr of linearized MC-YR, producing a tetrapeptide of Adda-Glu-Mdha-Ala (*m/z* 614) (product B) (Fig 6) and a tripeptide of Tyr-Masp-Arg (*m/z* 466) as the second products. The third enzyme peptidase went on cutting off the peptide bond between Adda and Glu in the linear tetrapeptide of Adda-Glu-Mdha-Ala to produce Adda as the third product (*m/z* 331) (product C) (Fig 7). Finally the fourth enzyme peptidase cleaved the peptide bond of Tyr-Masp in the linear tripeptide of Tyr-Masp-Arg, and Tyr (*m/z* 181) (product D) and dipeptide (Masp-Arg) (*m/z* 268) were produced (Fig 8). Generally, the biodegradation pathway of MC-YR was very similar to that of MC-RR and MC-LR in the first three steps we have reported previously [18,19], [28,29]. Here we firstly provided direct evidence that the tripeptide of Tyr-Masp-Arg could also be further biodegraded, and Tyr was produced as a biodegradation product of MC-YR (Fig 3 and 8). These findings might provide valuable evidence for further investigation on the biodegradation mechanism of MCs by *Sphingopyxis* sp. USTB-05. Whether the biodegradation pathways of MC-RR or MC-LR are also similar to that of MC-YR need further exploration, which may benefit both basic research and the elimination of MCs in lakes, reservoirs, and water treatment plants.

## Conclusion

Biodegradation is considered to be the most efficient and environment-friendly method for the reduction of MCs in natural eutrophic lakes and reservoirs. Based on the successful isolation of a promising bacterial strain *Sphingopyxis* sp. USTB-05 for the biodegradation of MCs, we successfully investigated its enzymatic biodegradation pathway toward MC-YR, which contains more complex Tyr in the ring structure of heptapeptide. Firstly, initial MC-YR of 14.8 mg/L was completely removed within 10 hr by CEs of *Sphingopyxis* sp. USTB-05 in the presence of 103.6 mg/L protein. Secondly, four biodegradation products of MC-YR were found over time on HPLC profiles at the wavelength of 238 nm and 230 nm, respectively. Thirdly, the biodegradation pathway of MC-YR by *Sphingopyxis* sp. USTB-05 was suggested. The first enzyme microcystinase was active in cleaving the target peptide bond between Adda and Arg of MC-YR and converting cyclic MC-YR to linear MC-YR as the first product. Then the second enzyme serine protease was found to cleave the target peptide bond between Ala and Tyr of linearized MC-YR, the tetrapeptide of Adda-Glu-Mdha-Ala and the tripeptide of Tyr-Masp-Arg were produced as the second products. The third enzyme peptidase then cut off the peptide bond between Adda and Glu in the linear tetrapeptide of Adda-Glu-Mdha-Ala to produce Adda as the third product. Finally the fourth enzyme peptidase cleaved the peptide bond of Tyr-Masp in the linear tripeptide of Tyr-Masp-Arg, producing Tyr. This study is crucial for both basic research and the removal of MCs from natural water source.
